# Prediction of high-flow nasal cannula outcomes at the early phase using the modified respiratory rate oxygenation index

**DOI:** 10.1186/s12890-022-02017-8

**Published:** 2022-06-13

**Authors:** Zhe Li, Chen Chen, Zhangjun Tan, Yulong Yao, Shunpeng Xing, Yan Li, Yuan Gao, Zhanqi Zhao, Yuxiao Deng, Mingli Zhu

**Affiliations:** 1 Department of Critical Care Medicine, Renji Hospital, School of Medicine, Shanghai Jiao Tong University, 160 Pujian Road, Shanghai, 200127 China; 2 Department of Biomedical Engineering, Fourth Military Medical University, Xi’an, China; 3 Institute of Technical Medicine, Furtwangen University, Villingen-Schwenningen, Germany

**Keywords:** High flow nasal cannula, Oxygen therapy, Acute respiratory failure, ROX index, Intubation

## Abstract

**Background:**

This study was designed to explore the early predictive value of the respiratory rate oxygenation (ROX) index modified by PaO_2_ (mROX) in high-flow nasal cannula (HFNC) therapy in patients with acute hypoxemia respiratory failure (AHRF).

**Method:**

Seventy-five patients with AHRF treated with HFNC were retrospectively reviewed. Respiratory parameters at baseline and 2 h after HFNC initiation were analyzed. The predictive value of the ROX (ratio of pulse oximetry/FIO_2_ to respiratory rate) and mROX (ratio of arterial oxygen /FIO_2_ to respiratory rate) indices with two variations by adding heart rate to each index (ROX-HR and mROX-HR) was evaluated.

**Results:**

HFNC therapy failed in 24 patients, who had significantly higher intensive care unit (ICU) mortality and longer ICU stay. Both the ROX and mROX indices at 2 h after HFNC initiation can predict the risk of intubation after HFNC. Two hours after HFNC initiation, the mROX index had a higher area under the receiver operating characteristic curve (AUROC) for predicting HFNC success than the ROX index. Besides, baseline mROX index of greater than 7.1 showed a specificity of 100% for HFNC success.

**Conclusion:**

The mROX index may be a suitable predictor of HFNC therapy outcomes at the early phase in patients with AHRF.

**Supplementary Information:**

The online version contains supplementary material available at 10.1186/s12890-022-02017-8.

## Background

Acute hypoxemia respiratory failure (AHRF) is a common cause of hypoxemia in patients admitted to the intensive care unit (ICU) and leads to high rates of mortality. High-flow nasal cannula (HFNC) therapy introduces low levels of airway pressure that increases end-expiratory lung volume; improves oxygenation, regional lung aeration [1–3], respiratory drive, and lung mechanics; and enhances CO_2_ removal [4–6].

Recently, HFNC has gained popularity among intensivists to manage patients with AHRF [7, 8]. However, in cases of HFNC failure, the delay of intubation could increase ICU mortality by 27.5% and prolong ventilator duration [9, 10]. Therefore, early predictors of HFNC outcomes to avoid intubation delay are desirable. High scores in the simplified Acute Physiology and Chronical Health Evaluation II (APACHE II), high sensitive C-reactive protein levels, high heart rate (HR) to saturation of pulse oxygen (SpO_2_) ratio, and tachycardia during HFNC therapy were indirectly correlated with remedial intubation after HFNC therapy [11–14].

The respiratory rate oxygenation (ROX) index, which is the ratio of SpO_2_/fraction of inspired oxygen (FiO_2_) to respiratory rate (RR), is a convenient and effective index for predicting HFNC failure in patients with AHRF caused by pneumonia [15]. Besides, researchers have examined whether the ROX index can be further improved by incorporating other clinical parameters. A retrospective study has proposed that ROX index values of more than 5.98 with FiO_2_ of less than 0.59 at 8 h after HFNC therapy were associated with a less risk of mechanical ventilation (MV) in patients with AHRF [16]. Goh et al. have verified that ROX index with heart rate (ROX-HR: ROX/HR*100) values of more than 6.80 were significantly associated with a lower risk of intubation 10 h after HFNC in patients with AHRF [17].

A few small studies have attempted to determine early parameters to avoid intubation delays; however, the clinical challenge is that no particularly good parameters have been concluded so far [18, 19]. SpO_2_ of the ROX index was monitored using near-infrared light and could be influenced by body temperature, acid–base status, hypoperfusion, and hemoglobin, among others; moreover, there is discrepancy between SpO_2_ and the actual oxygen status [20, 21]. Karim and Esquinas have elaborated the physiological correlation between SpO_2_ and arterial oxygen partial pressure (PaO_2_), and suspected PaO_2_ may better reflect the true oxygenation status of patients [22]. Therefore, they proposed that the ROX index modified by incorporating PaO_2_ (mROX index: PaO_2_/FiO_2_/RR) may predict HFNC outcomes better. However, up to now, limited data are available on the effectiveness of the mROX index, and the potential of the mROX index achieving a better predictive value to avoid intubation at an early phase after HFNC therapy remains unclear. Therefore, this study was designed to explore the ability of the mROX index to predict HFNC therapy outcomes in patients with AHRF at an early phase.

## Materials and methods

### Study population and ethical approval

A retrospective cohort study involving patients with AHRF receiving HFNC therapy was conducted. Consecutive patients admitted to the surgical ICU of Renji Hospital from January 2020 to June 2020 were enrolled in this study. This study was conducted in accordance with the amended Declaration of Helsinki (as revised in 2013) and approved by the Ethics Committee of Renji Hospital, School of Medicine, Shanghai Jiao Tong University (ky2020-059).

Adult patients (above 18 years) admitted to the ICU due to AHRF receiving HFNC therapy were included. Patients with incomplete data, those who were discharged from the hospital after giving up further treatment, and those within the perinatal period were excluded.

### Study definition

#### Diagnosis of AHRF

AHRF is defined as having an RR of more than 25 breaths/min and a P/F ratio of less than 300 mmHg on an oxygen device delivering ≥ 10 L/min, in the absence of chronic respiratory failure and hypercapnia.

#### HFNC therapy

HFNC therapy was implemented using Optiflow (Fisher and Paykel Healthcare, East Tamaki, New Zealand) or HFNC module in V300 or V500 (Dräger Medical, Lübeck, Germany) associated with humidification in this study. The initial setting was FiO_2_ of 100% at a flow rate of 50–60L/min. Flows or FiO_2_ was adjusted as appropriate, with a target SpO_2_ of ≥ 92%. HFNC parameters were adjusted based on clinical judgment; patients were switched to nasal tube for oxygen delivery when the flow is less than 20 L/min and FiO_2_ is less than 30%.

#### HFNC success

HFNC success was defined as improvement of respiratory status without requiring intubation for MV during ICU stay.

#### Criteria of intubation for MV

Patients with AHRF who received HFNC therapy were considered, and intubation for MV was performed when a patient meets at least one of the following *criteria*: failure to achieve correct oxygenation (PaO_2_ of less than 60 mmHg despite HFNC flow of ≥ 30L/min and FiO_2_ of 100%), respiratory acidosis (PaCO_2_ of more than 50 mmHg with pH of less than 7.25), RR of more than 40 breaths/min, significant hemodynamic instability (cardiac arrest/arrhythmias, severe hemodynamic instability, or norepinephrine of more than 0.1 μg/kg/min), deterioration in neurological status (Glasgow Coma Scale (GCS) of less than 12), and inability to clear secretions.

#### Subjects and outcomes

The primary outcome was the number of patients who were not intubated during ICU stay; moreover, the durations of HFNC therapy and ICU stay were analyzed.

#### Data collection

Demographic data, etiology of AHRF and ICU admission, APACHE II score, respiratory parameters, blood gas parameters on admission and 2 h after HFNC initiation, and intubation situation were collected. The patients were divided into the HFNC success and HFNC failure groups.

#### mROX index calculation

The ROX (1) and mROX (2) indices with two variations by adding heart rate ((ROX-HR) (3) and mROX-HR (4)) were calculated as follows:

1 $${\text{ROX}} = {\raise0.7ex\hbox{${{\text{SpO}}_{2} }$} \!\mathord{\left/ {\vphantom {{{\text{SpO}}_{2} } {}}}\right.\kern-\nulldelimiterspace} \!\lower0.7ex\hbox{${}$}}\left( {{\text{FiO}}_{2} \cdot RR} \right)$$

2 $${\text{mROX}} = {\raise0.7ex\hbox{${{\text{PaO}}_{2} }$} \!\mathord{\left/ {\vphantom {{{\text{PaO}}_{2} } {\left( {{\text{FiO}}_{2} \cdot RR} \right)}}}\right.\kern-\nulldelimiterspace} \!\lower0.7ex\hbox{${\left( {{\text{FiO}}_{2} \cdot RR} \right)}$}}$$

3 $${\text{ROX}} - {\text{HR}} = \left[ {{\raise0.7ex\hbox{${{\text{SpO}}_{2} }$} \!\mathord{\left/ {\vphantom {{{\text{SpO}}_{2} } {{\text{FiO}}_{2} \cdot RR \cdot HR}}}\right.\kern-\nulldelimiterspace} \!\lower0.7ex\hbox{${{\text{FiO}}_{2} \cdot RR \cdot HR}$}}} \right]{*}100$$

4 $${\text{mROX}} - {\text{HR}} = \left[ {{\raise0.7ex\hbox{${{\text{PaO}}_{2} }$} \!\mathord{\left/ {\vphantom {{{\text{PaO}}_{2} } {\left( {{\text{FiO}}_{2} \cdot RR \cdot HR} \right)}}}\right.\kern-\nulldelimiterspace} \!\lower0.7ex\hbox{${\left( {{\text{FiO}}_{2} \cdot RR \cdot HR} \right)}$}}} \right]{*}100$$

#### Statistical analysis

Continuous data are expressed as means ± standard deviations for normally distributed data and medians (25th percentile (P25) and 75th percentile (P75)) for non-normally distributed data. The chi-square test or Fisher’s exact test was used to compare categorical variables. Non-normally distributed continuous variables were compared using the Mann–Whitney U-test. Logistic regression analysis was used to determine the variables associated with HFNC treatment success.

The predictive values of the ROX, mROX, ROX-HR, and mROX-HR indices at baseline and 2 h after HFNC initiation were expressed as the area under the receiver operating characteristic curve (AUROC). Decision curve analysis was used to assess the net benefit. The optimal cutoff values were determined using Youden’s Index. Differences with* p* values of less than 0.05 were considered statistically significant. All statistical analyses were performed using Statistical Package for the Social Sciences (version 19.0; IBM Corp., Armonk, NY, USA).

## Results

### Patient population and HFNC outcomes

**Fig. 1:**
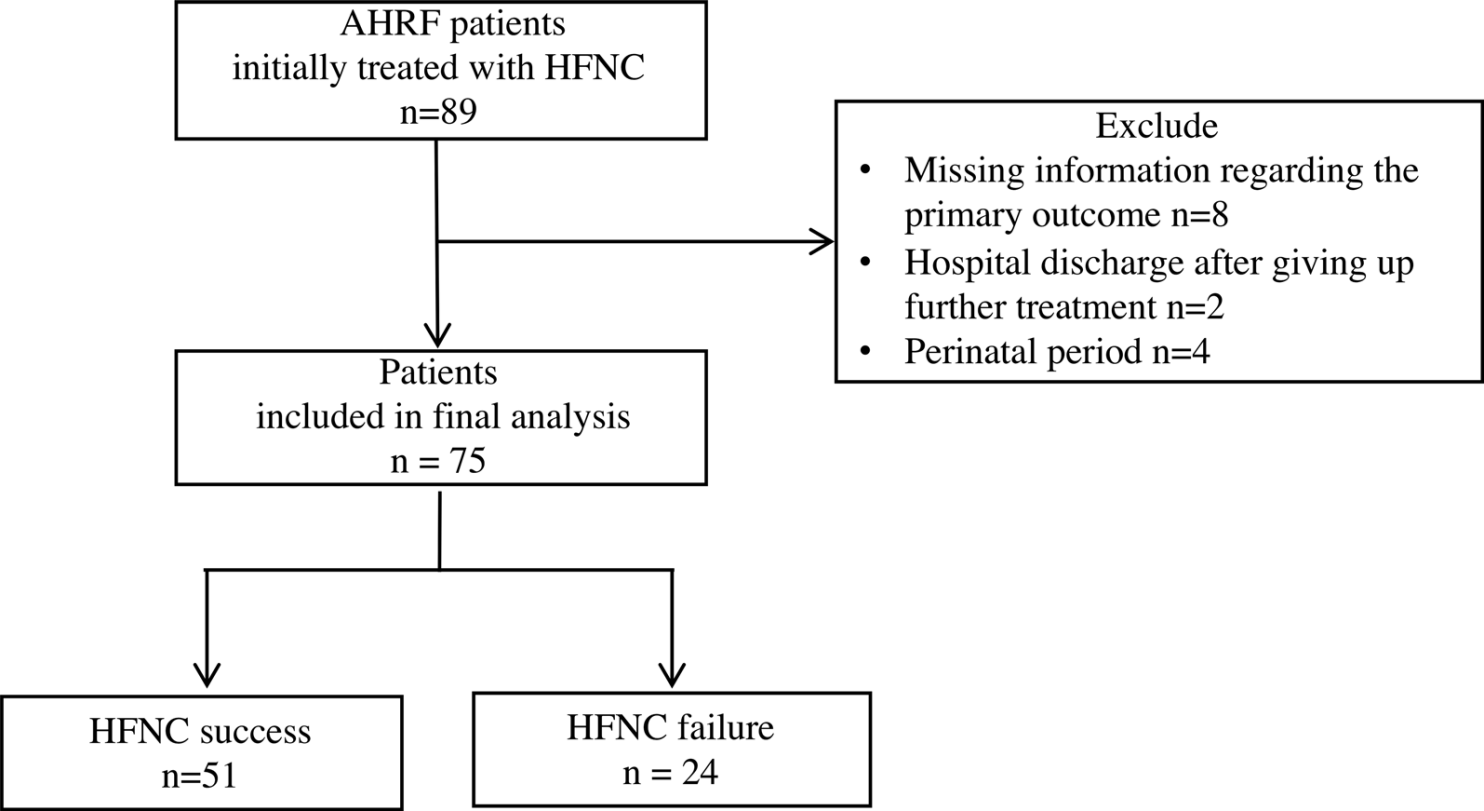
Flowchart of the study process

Among the 89 patients with AHRF initially treated with HFNC therapy who were admitted to our center during the study period, eight were excluded for missing information regarding the primary outcome, two were excluded because they were discharged from the hospital after giving up further treatment, and four were excluded because they were in the perinatal period (Fig. 1).

Finally, 75 patients were eligible for analysis. The demographic data of the study population are presented in Table 1. Twenty-four patients (32%) were intubated after HFNC therapy for MV, the earliest intubation was performed at 3 h after HFNC therapy initiation. The HFNC failure group had a longer duration of ICU stay than the HFNC success group (42 d (16.3–68.5)vs. 11 d(5–23); *p* < 0.001) and higher ICU mortality (54.2% vs. 2.0%; *p* < 0.001). Hypertension was the most common complication in the study subjects. Noncardiogenic pulmonary edema (31.4%) was the primary etiology for AHRF in the HFNC success group, whereas viral pneumonia due to coronavirus disease 2019 (COVID-19) (54.2%) was the primary etiology for AHRF in the HFNC failure group. The duration of HFNC therapy in the HFNC failure group was shorter than that in the HFNC success group (22.0 h (7.3–58.3) vs. 54 h (22–127); *p* < 0.001). No other demographic and clinical characteristics showed significant differences between the two groups (Table 1). Complete vital signs at the point of HFNC failure were captured in 10 patients from the HFNC failure group and summarized in Additional file 1: Table S1.

**Table 1:** Demographic and clinical characteristics of the study population (n = 75)

	HFNC-success (n = 51)	HFNC-failure (n = 24)	*p *value
Age, years (mean ± SD, years)	62.9 ± 2.0	69.3 ± 2.4	0.062
Male gender [*n*(%)]	39 (76.5)	18 (75.0)	0.889
APACHE II*[*M*(*P*_25_, *P*_75_)]	14 (11–22)	15 (13–18)	0.968
Comorbidities [*n*(%)]			
Moderate to severe CKD	6 (11.8)	7 (29.2)	0.100
Congestive heart failure	8 (15.7)	7 (29.2)	0.219
Chronic respiratory disease	5 (9.8)	4 (16.7)	0.455
Diabetes	11 (21.6)	8 (33.3)	0.274
Hypertension	16 (31.4)	10 (41.7)	0.382
Nervous system disease	5 (9.8)	6 (25.0)	0.158
Immunocompromised host	6 (11.8)	3 (12.5)	1.000
Primary etiology for respiratory failure [*n*(%)]			
Non‐COVID pneumonia	13 (25.5)	5 (20.8)	0.660
COVID‐19 pneumonia	14 (27.5)	13 (54.2)	0.025
Noncardiogenic pulmonary edema*	16 (31.4)	5 (20.8)	0.343
Cardiogenic pulmonary edema	7 (13.7)	0 (0)	0.089
Pulmonary embolism	1 (2.0)	1 (4.2)	0.541
Duration of HFNC [*M*(*P*_25_, *P*_75_), h]	54.0 (22.0–127.0)	22 .0(7.3–58.3)	0.001
ICU stay duration [*M*(*P*_25_, *P*_75_), d]	11.0 (5.0–23.0)	42 .0(16.3–68.5)	0.000
ICU mortality [*n*(%)]	1 (2.0)	13 (54.2)	0.000

Annotation: APACHE II, Acute Physiology and Chronical Health Evaluation II; HFNC, high-flow nasal cannula; CKD, chronic kidney disease; MV, mechanical ventilation; ICU, intensive care unit. COVID-19, coronavirus disease 2019.

* acute respiratory distress syndrome due to acute pancreatic and extra-pulmonary infections

### Variables at baseline and 2 h after HFNC

Among the baseline variables, the HFNC success group had lower baseline HR (101.8 ± 18.3 vs. 116.5 ± 16.6, beats/min; *p* = 0.001), RR (24.7 ± 4.9 vs. 27.9 ± 5.8, counts/min; *p* = 0.015), and lactate (2 ± 1.2 vs. 2.7 ± 1.9, mmol/L; *p* = 0.044) than the HFNC failure group. Other variables did not show significant differences between the two groups (Table 2). Among the variables under study 2 h after HFNC therapy initiation, FiO_2_ was lower (0.55 (0.50–0.60) vs. 0.69 (0.60–0.89); *p* = 0.001) and the PF ratio was higher (178.0 (126.0–251.7) vs. 95.7 ( 68.6–131.7); *p* < 0.001) in the HFNC success group than those in the HFNC failure group.

**Table 2:** Variables at baseline and 2 h after HFNC therapy initiation

	HFNC success (n = 51)	HFNC failure (n = 24)	*p *value
Baseline HR [mean ± SD, bpm]	102 ± 18	116 ± 17	0.001
Baseline RR [mean ± SD, cpm]	25 ± 5	28 ± 6	0.015
Baseline FiO_2_ [mean ± SD]	0.5 ± 0.2	0.5 ± 0.2	0.641
Baseline SpO_2_ [mean ± SD, %]	95.0 ± 4.0	93.2 ± 6.1	0.140
Baseline PaO_2_ [*M*(*P*_25_, *P*_75_), mmHg]	70.0 (61.0–84.0)	69.8 (60.4–75.4)	0.222
Baseline PaCO_2_ [mean ± SD, mmHg]	37.4 ± 7.7	34.2 ± 6.5	0.080
Baseline PF ratio [*M*(*P*_25_, *P*_75_)]	142.5 (123.6–188.4)	138.0 (114.5–171.5)	0.214
Baseline lactate [mean ± SD, mmol/l]	2.0 ± 1.2	2.7 ± 1.9	0.044
2 h HR [mean ± SD,bpm]	99 ± 17	113 ± 16	0.001
2 h RR [*M*(*P*_25_, *P*_75_),bpm]	21 (19–25)	27 (23–35)	0.001
2 h FiO_2_ [*M*(*P*_25_, *P*_75_)]	0.55 (0.50–0.60)	0.69 (0.60–0.89)	0.001
2 h Flow on HFNC [mean ± SD, L/min]	49.7 ± 7.7	52.1 ± 8.2	0.226
2 h SpO_2_ [*M*(*P*_25_, *P*_75_)]	98.0 (96.0–100.0)	95.0 (89.5–97.8)	0.002
2 h PaO_2_ [mean ± SD, mmHg]	106.7 ± 41.5	75.3 ± 25.2	0.001
2 h PaCO_2_ [mean ± SD, mmHg]	35.9 ± 8.4	34.2 ± 6.0	0.386
2 h PF ratio [*M*(*P*_25_, *P*_75_)]	178.0 (126.0–251.7)	95.7(68.6–131.7)	0.000
2 h lactate [*M*(*P*_25_, *P*_75_), mmol/l]	1.4 (1.0–2.0)	2.3(1.7–3.6)	0.001

Annotation: HR, heart rate; RR, respiratory rate; FiO_2_, fraction of inspiration O_2_; SpO_2_,saturation of pulse oxygen ; PaO_2_, arterial oxygen partial pressure; PaCO_2_, arterial carbon dioxide partial pressure; PF ratio, PaO_2_/FiO_2_; cpm, counts per minute; bpm, beats per minute; P_25_, 25th percentile; P_75_, 75th percentile; HFNC, high-flow nasal cannula

### The relationship between SpO_2_ and PaO_2_ in HFNC success and failure group

**Fig. 2:**
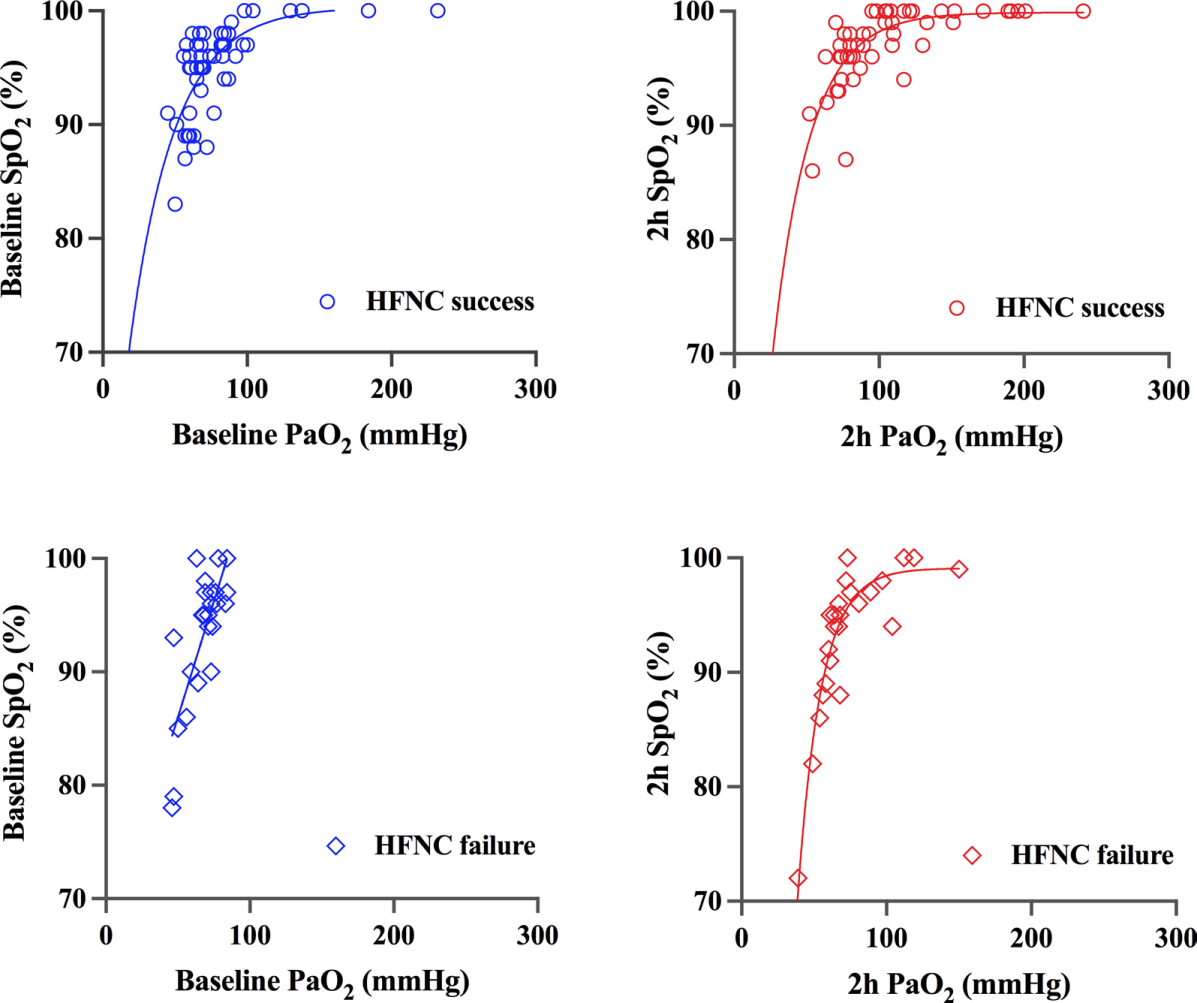
Scatter plots of SpO_2_ over PaO_2_ in the HFNC success and failure groups. Left: data at baseline; right: data at 2 h after HFNC initiated. Top: HFNC success group; bottom: HFNC failure group

The plots of SpO_2_ over PaO_2_ in all patients were illustrated in Fig. 2. More patients achieved the SpO_2_ value of 100% at 2 h after HFNC therapy initiation compared to baseline (21vs.9). Most of these patients were in the HFNC success group (18 vs.3).

### The ROX, mROX, ROX-HR, and mROX-HR indices predict HFNC success

**Fig. 3:**
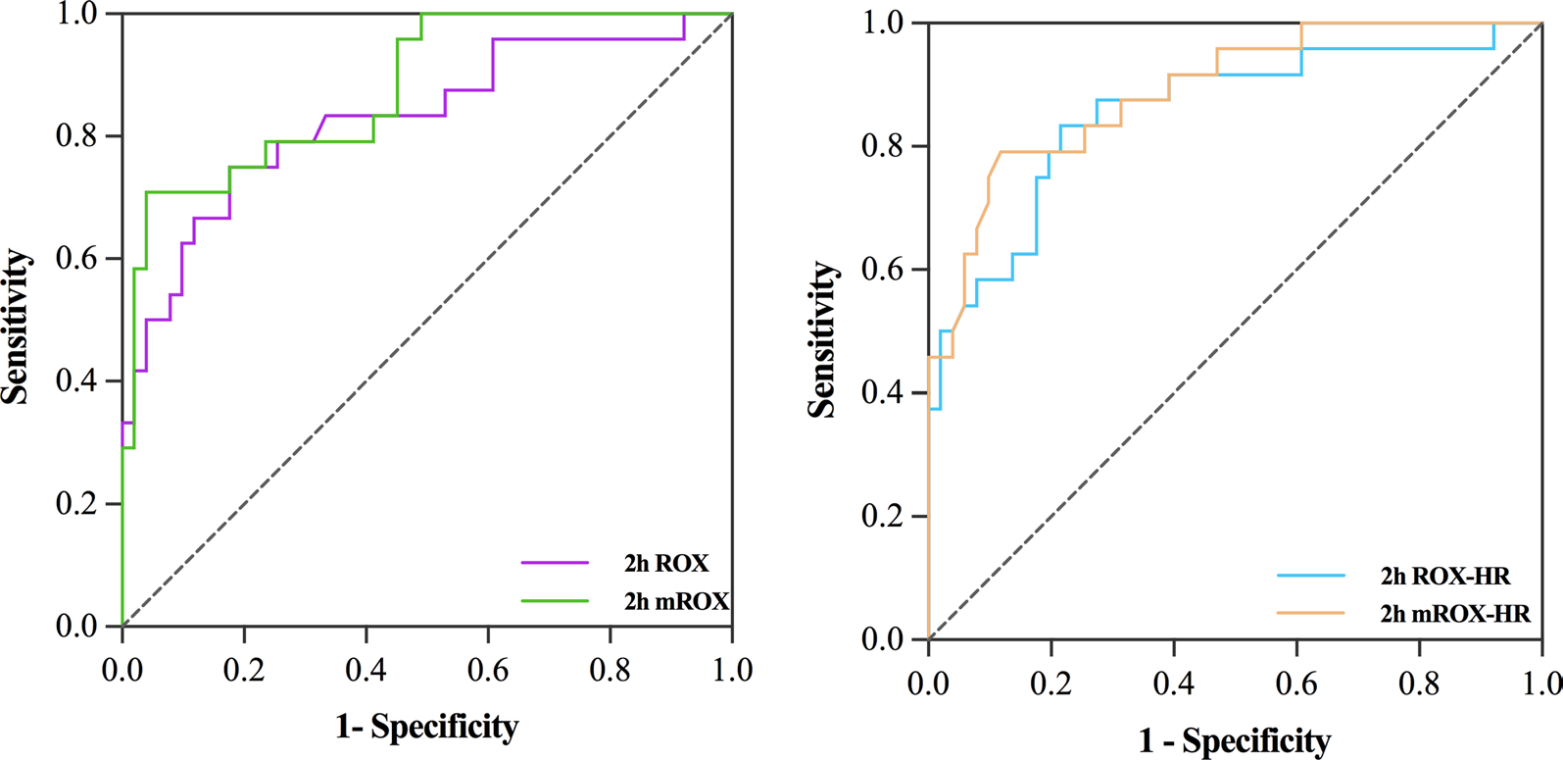
The ROC curve of the ROX, mROX, ROX-HR, and mROX-HR indices for predicting HFNC success. ROX, SpO_2_/FiO_2_ (%) to RR (breaths/min); mROX, PaO_2_/FiO_2_(%) to RR(breaths/min); ROX-HR,ROX/HR*100; mROX-HR, mROX /HR*100; ROC: operating characteristic curve

**Fig. 4:**
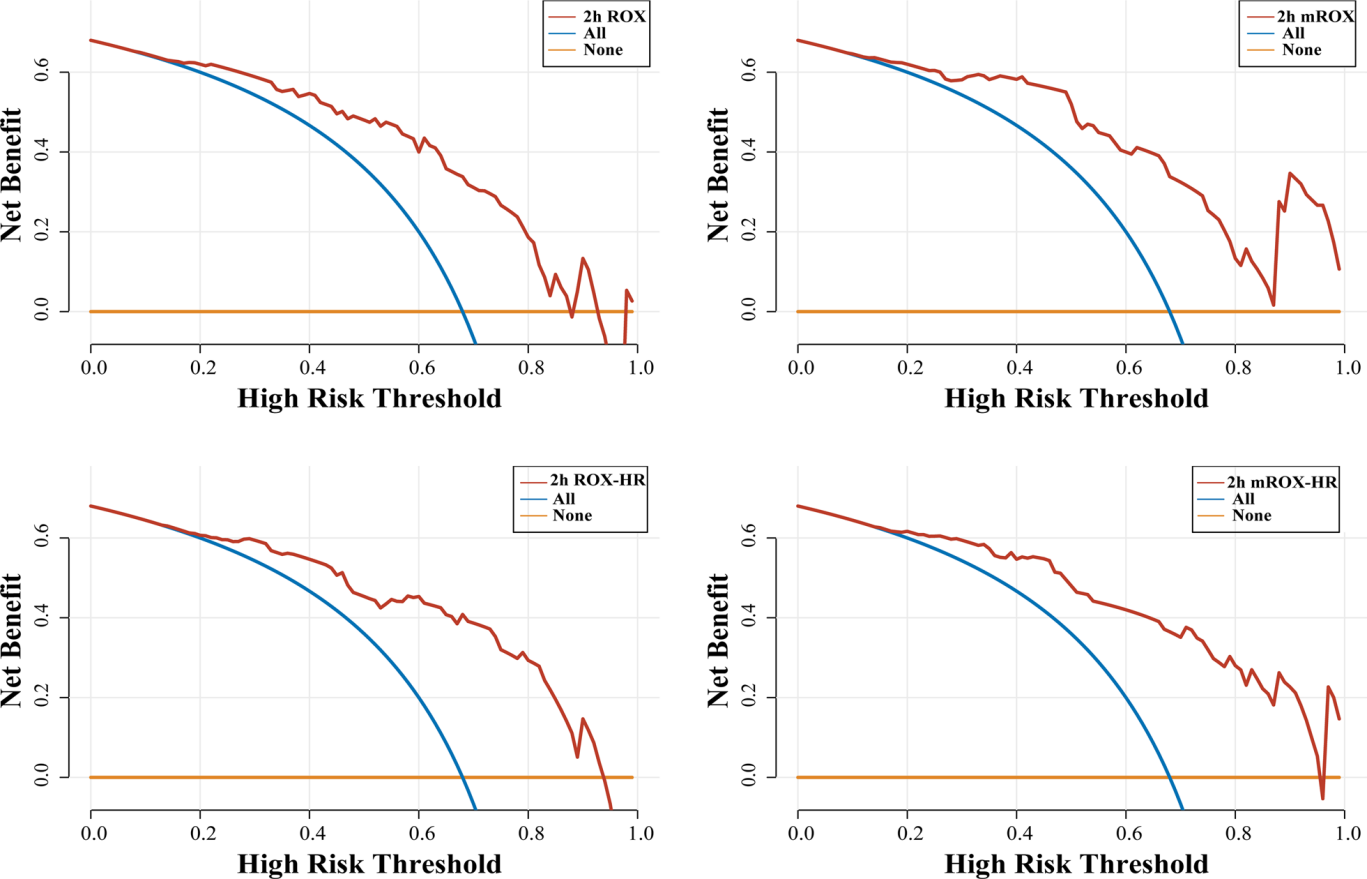
The decision analysis curves of ROX (top left), mROX (top right), ROX-HR (bottom left), and mROX-HR (bottom right) indices at 2 h after HFNC therapy initiation

The baseline mROX index values in the HFNC success and failure groups were 6.1 (4.3–9.0) and 5.2 (4.0–6.5), respectively, and the 2 h mROX index values were 8.7 (6.0–12.0) and 3.7 (2.6–5.5), respectively. The ROX, mROX, ROX-HR, and mROX-HR indices were lower in the HFNC failure group both at baseline and 2 h after HFNC therapy initiation than those in the HFNC success group. The mROX and mROX-HR indices had greater OR value associated with HFNC success than those of ROX and ROX-HR at 2 h after HFNC therapy initiation. Besides, the baseline ROX index was the only index that did not show a significant difference between the two groups (Table 3). The ROX, mROX, ROX-HR, and mROX-HR indices at 2 h after HFNC therapy initiation performed better at predicting HFNC success than those at baseline with AUROC values being over 0.8. 2 h mROX (0.878, 95% CI: 0.795–0.961) had greater AUROC than 2 h ROX (0.829, 95% CI:0.721–0.937). The same results were found in 2 h mROX-HR (0.891, 95%CI: 0.812–0.969) and 2 h ROX-HR (0.855, 95% CI:0.757–0.954). Furthermore, 2 h mROX-HR had the largest AUROC for predicting HFNC success (Fig. 3). Nevertheless, the AUROC values between mROX and ROX indices and between mROX-HR and ROX-HR at 2 h were not statistically significant (Table 4). The decision curve analysis showed that the 2 h mROX yielded higher net benefits in predicting HFNC success than 2 h ROX, similar results was also observed in 2 h mROX-HR and 2 h ROX-HR indices (Fig. 4). According to the highest Youden’s index, the cutoff value of more than 4.3 for 2 h mROX index had the highest sensitivity (96.1%), and the cutoff value of more than 7.1 for baseline mROX index had highest specificity (100%) for predicting HFNC success.

**Table 3:** The ROX, mROX, ROX-HR, and mROX-HR indices predict HFNC success

	HFNC-success (n = 51)	HFNC-failure (n = 24)	*p* value	OR value	95% CI
Baseline ROX [M(*P*_25_, *P*_75_)]	7.5 (6.0–10.6)	7.4 (5.4–8.4)	0.061	1.968	0.970–3.994
Baseline mROX [M(*P*_25_, *P*_75_)]	6.1 (4.3–9.0)	5.2 (4.0–6.5)	0.012	2.631	1.233–5.613
Baseline ROX-HR [M(*P*_25_, *P*_75_)]	7.6 (5.3–11.7)	6.0 (5.0–7.3)	0.013	3.013	1.259–7.210
Baseline mROX-HR [M(*P*_25_, *P*_75_)]	6.8 (4.2–9.8)	4.2 (3.4–5.7)	0.005	3.984	1.526–10.402
2 h ROX [mean ± SD]	8.3 ± 2.5	5.4 ± 2.3	0.001	5.954	2.434–14.613
2 h mROX [M(*P*_25_, *P*_75_)]	8.7 (6.0–12)	3.7 (2.6–5.5)	0.001	10.816	3.373–34.689
2 h ROX-HR [mean ± SD]	8.9 ± 3.7	4.9 ± 2.6	0.001	8.696	2.797–27.038
2 h mROX-HR [M(*P*_25_, *P*_75_)]	8.9 (5.8–12.6)	3.6 (2.3–4.1)	0.001	15.039	3.758–60.190

Annotation: ROX, SpO_2_ /FiO_2_ (%) to RR (breaths/min); mROX, PaO_2_/FiO_2_(%) to RR(breaths/min); ROX-HR,ROX /HR*100; mROX-HR, mROX/HR*100; OR, odds ratio; CI, Confidence interval

**Table 4:** Predictive values of the ROX, mROX, ROX-HR, and mROX-HR indices for HFNC success

Indices	AUROC	95% CI	Cutoff	Sensitivity	Specificity	*p* value
Baseline mROX	0.661	0.540–0.782	7.1	0.431	1.00	
Baseline ROX-HR	0.676	0.557–0.796	7.9	0.471	0.917	
Baseline mROX-HR	0.722	0.610–0.835	6.7	0.510	0.958	
2 h ROX	0.829	0.721–0.937	6.2	0.824	0.750	0.1997
2 h mROX	0.878	0.795–0.961	4.3	0.961	0.708	
2 h ROX-HR	0.855	0.757–0.954	5.8	0.784	0.833	0.2141
2 h mROX-HR	0.891	0.812–0.969	4.1	0.902	0.792	

Annotation: AUROC, area under the receiver operating characteristic curve; CI: confidence interval; ROX, SpO_2_ /FiO_2_ (%) to RR (breaths/min);mROX, PaO_2/_FiO_2_(%) to RR(breaths/min);ROX-HR,ROX /HR*100 ;mROX-HR,mROX /HR*100

### The ROX, mROX, ROX-HR, and mROX-HR indices for HFNC success in COVID-19 pneumonia and Non-COVID-19 subgroups

**Fig. 5:**
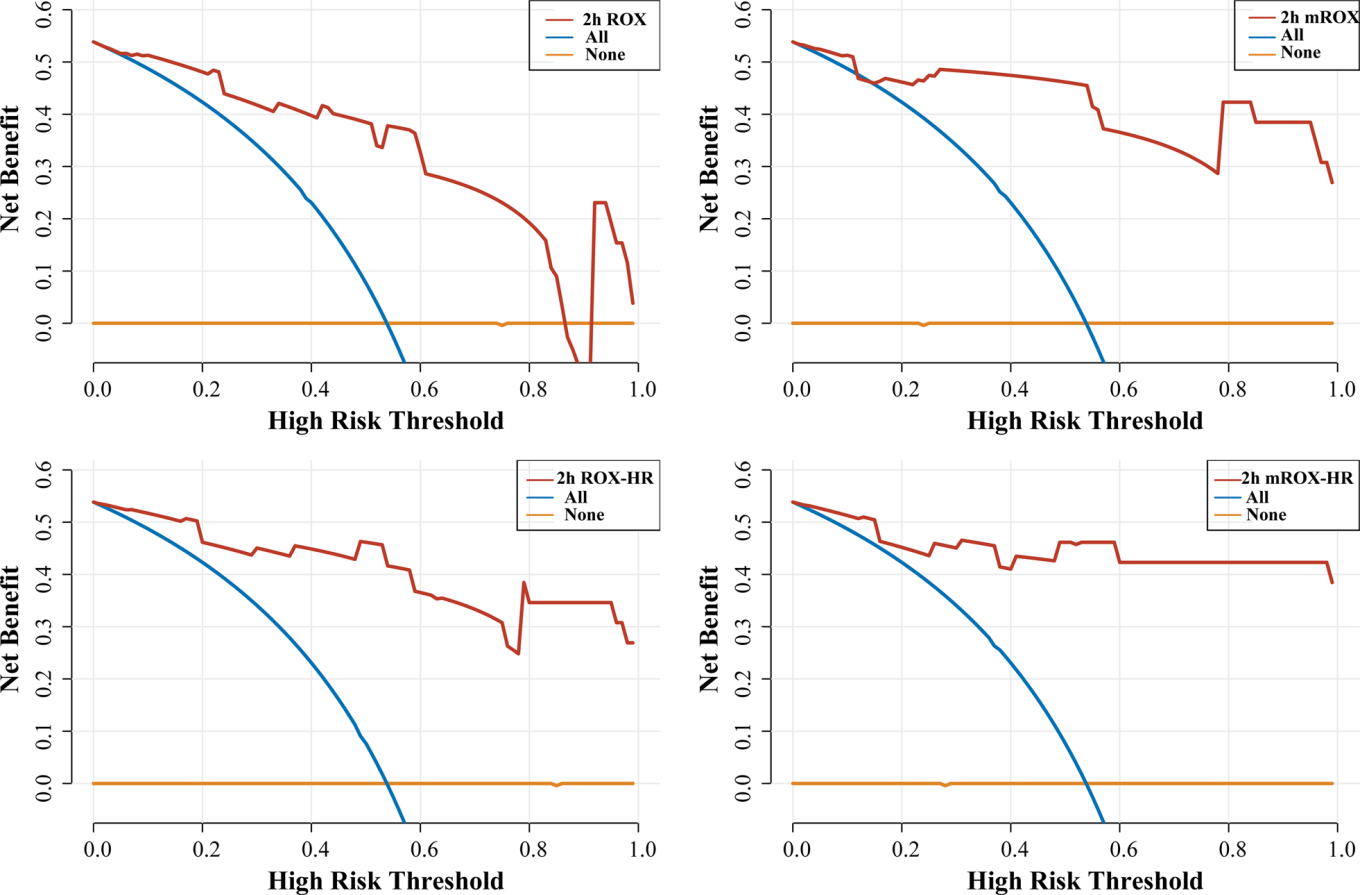
The decision analysis curves of ROX (top left), mROX (top right), ROX-HR (bottom left), and mROX-HR (bottom right) indices at 2 h after HFNC therapy initiation in COVID-19 pneumonia

The ROX, mROX, ROX-HR, and mROX-HR indices at 2 h after HFNC therapy initiation have greater AUROC value by at least 0.14 at predicting HFNC success in COVID-19 pneumonia patients than those in the Non-COVID-19 patients. Besides, 2 h mROX (0.952, 95% CI: 0.790–0.998) showed a higher degree of AUROC difference than 2 h ROX (0.911, 95% CI: 0.732–0.986) in COVID-19 pneumonia patients than that in the Non-COVID-19 patients. The 2 h mROX-HR had a higher AUROC values than 2 h Rox-HR in Non-COVID-19 pneumonia patients, but no difference in COVID-19 patients, Nevertheless, the AUROC value between these indices was not statistically significant (Table 5). Further, decision curve analysis of COVID-19 pneumonia subgroup revealed that the 2 h mROX index yielded higher net benefits in predicting HFNC success than 2 h ROX, similar situation was also observed for 2 h mROX-HR and 2 h ROX-HR indices (Fig. 5).

**Table 5:** Predictive values of the ROX, mROX, ROX-HR, and mROX-HR indices for COVID-19 pneumonia and non-COVID-19 patients

Indices	COVID-19 pneumonia		Non-COVID-19		*P* value
	AUROC	95% CI	AUROC	95% CI	
2 h ROX	0.911	0.732–0.986	0.756	0.612–0.867	0.1514
2 h mROX	0.952	0.790–0.998	0.806	0.668–0.905	0.0749
2 h ROX-HR	0.958	0.799–0.999	0.775	0.633–0.882	0.0523
2 h mROX-HR	0.958	0.799–0.999	0.82	0.684–0.915	0.0742

Annotation: AUROC, area under the receiver operating characteristic curve; CI: confidence interval; ROX, SpO_2_ /FiO_2_ (%) to RR (breaths/min); mROX, PaO_2_/FiO_2_(%) to RR (breaths/min); ROX-HR, ROX/HR*100; mROX-HR, mROX /HR*100

## Discussion

In this retrospective observational study, we found that the ROX index modified by incorporating PaO_2_ may perform better at predicting HFNC therapy outcomes in patients with AHRF 2 h after HFNC therapy initiation.

### Prediction value of mROX index

To our knowledge, the role of baseline ROX and mROX indices in predicting HFNC outcomes has not yet been discussed. Our results revealed that baseline mROX index of greater than 7.1 showed a specificity of 100% for HFNC success. While baseline ROX index didn’t differ in success and failure group in our study. Patients with AHRF with baseline mROX index value of more than 7.1 seem to be in a safe range after HFNC initiation with extremely low possibility of intubation. Combining mathematical calculations with clinical practice, assuming that the patient’s RR is 25 bpm with FiO_2_ of 0.7, SaO_2_ must reach 125 mmHg to get mROX index value greater than 7.1; that is, these patients have relatively acceptable oxygenation status. The observation intensity and period of these patients could be relatively relaxed by clinicians.

The ROX index combines the best markers of patients’ respiratory status in a single value. Compared with patients with pneumonia from previous studies [15, 16, 23], 2 h ROX index had a better predictive value for HFNC success in this study. Besides, 2 h mROX had slightly greater AUROC of 0.05 than 2 h ROX, indicating that the mROX index has a better potential of predicting HFNC success at an early phase. Furthermore, our cutoff value for HFNC success was 4.3 (sensitivity, 96.1%; specificity, 70.8%) for 2 h mROX index and 6.2 (sensitivity, 82.4%; specificity, 75.0%) for 2 h ROX index. These results further suggest that intensivists should use the aforementioned cutoff value of the mROX index for predicting HFNC success, while the ROX index should be used for predicting intubation.

### PaO_2_ reflects ture oxygenation state

The ROX index is convenient and noninvasive; however, it has limitations in using SpO_2_ to represent oxygenation situation. First, the upper limit of SpO_2_ is 100%, which cannot reflect that oxygen worsens under high PaO_2_ [24]. Besides, the relationship between SpO_2_ and PaO_2_ is not linear [25]. Once SpO_2_ falls below 90% and oxygen status fluctuates greatly, based on this pathophysiology, SpO_2_ is at risk of failing to recognize respiratory deterioration [26]. In addition, a shift of the oxygen-hemoglobin dissociation curve, which is common in critically ill patients, affects the physiologically curvilinear relationship between SpO_2_ and PaO_2_. PaCO_2_ is reported as one of the most important factors affecting the oxygen dissociation curve[27]. In our patients, a lower PaCO_2_ value was observed in the failure group than that in the success group, which induced a leftward shift in the oxygen dissociation curve and resulted in an overestimated oxygenation state with the measured SpO_2_ values. These may partly explain why the ROX index required 12 h to achieve the highest predictive value for HFNC success, and the mROX index showed higher AUROC and sensitivity 2 h after HFNC therapy initiation. Moreover, as mentioned in the Introduction section, SpO_2_ is influenced by pathophysiological changes frequently occurring in critically ill patients. Our mROX index modified by incorporating PaO_2_ was validated to have a better predictive value for HFNC success at an early phase, which agrees with the notion that “PaO_2_ reflects the true state of oxygenation” by Karim and Esquinas [22]. However, the acquisition of PaO_2_ requires invasive arterial blood sampling and can produce additional cost of testing. According to clinical practice for critically ill patients, arterial blood gas analysis is a common and necessary bedside test recommended to be tested immediately after admission to the ICU and 1–2 h after adjusting respiratory treatment strategies [28, 29]. Therefore, 2 h PaO_2_ can be obtained conveniently without additional invasive procedures and costs in patients with AHRF admitted to the ICU.

### Variables that affect ROX index stability

As mentioned above, the AUROC of 2 h ROX in this study was higher than that of Roca (0.829 vs. training 0.602, validation 0.679). We suggest that this is due to the differences between medical and surgical care units and etiologies of AHRF. This study included perioperative patients with all-cause AHRF. Although pneumonia is the most frequent cause of AHRF, our results provide some information on the performance of the mROX and ROX indices in other etiologies of AHRF.

Beside, our cutoff value of 6.2 for 2 h ROX is also higher than Roca’s 4.88, a cutoff value similar to 5.8 in Goh’s study involving patients with AHRF admitted to emergency and medical ICUs [17]. The absolute value of the ROX index at the same time point varies in different studies. During clinical HFNC therapy, each change in the patients’ respiratory patterns and RRs could influence the ROX index, even with the same SpO_2_ and FiO_2_ [30]. Mauri had further shown that the ROX index value in patients with AHRF could be significantly impacted by the set flow rate [31]. A dynamic evaluation of the ROX index rather than single-point absolute value among patients with AHRF with ROX index values between the boundaries of below 3.85 for failure and above 4.88 for success was proposed to remedy ROX index instability [32]. Patients who had a dynamic reduction in ROX value of 0.5 between 2, 6, and 12 h after HFNC therapy initiation are suggested to consider intubation [8]. Dynamic sensitive assessment of the ROX index using the set flow rate of HFNC might be used to distinguish more severe patients at a higher risk of failure [19, 33]. In addition to dynamic assessment of the ROX index, the mROX index reflects the true state of oxygenation; timely evaluation of the mROX index for patients who had a decreased ROX index could help facilitate clinical decision and minimize intubation delay.

In addition, the mROX-HR index 2 h after HFNC therapy initiation had the highest AUROC (0.891) for predicting HFNC success compared with other indices, which agrees with previous results by Goh [17], who reported that 10 h ROX-HR index had higher AUROC for predicting HFNC success than the ROX index (0.739 vs. 0.723), indicating that incorporating HR could improve the predictive value of the ROX and mROX indices. Our cutoff value of 5.8 for 2 h ROX-HR for predicting HFNC success was less than 6.8 of Goh; the reason for this difference may be because we did not exclude patients with arrhythmia and cardiogenic pulmonary edema and the average HR in this study was higher. Another reason may be that HR would also be affected by multiple factors, such as body temperature and vasoactive and sedative drugs. Therefore, incorporating HR may be a noninvasive parameter that can help improve the predictive value of the mROX index; however, the application must consider factors that affect HR.

### Computational confines of ROX and mROX indices

Despite disease type differences, in patients with SpO_2_ of 95%, the ROX index is unlikely to drop below 4.88 with FiO_2_ up to 0.5 unless the RR is greater than 40. Tatkov S noticed that this situation deviates from clinical practice [30, 34]. Although the difference between calculation and reality cannot be avoided, the mROX index in this study is consistent with clinical practice and could be applied more widely in patients with AHRF (Additional file 1: Fig. S1).

### Limitations

This study explored the predictive value of 2 h mROX and baseline mROX indices for HFNC outcomes in perioperative patients with AHRF. However, this study has some limitations. First, it is a single-center retrospective observational study, in the process of data collection, we found that some information was missing which may lead to bias, such as the vital signs at the time point of intubation. Therefore, our results should be interpreted with caution. Further validation in prospective, large-cohort, and multicenter studies are warranted. Second, since the mROX calculations require arterial blood gas analysis, we did not collect the mROX index at continuous time points after HFNC therapy initiation. Third, a separate validation was missing for the ROX/mROX thresholds, which should be further evaluated in future prospective studies. Lastly, we did not evaluate the predictive value of the mROX index in patients receiving HFNC after extubation or patients with ARF with hypercapnia, which could be directions for future studies.

## Conclusion

The results of this study suggest that the mROX index holds potential to be a parameter for early prediction of HFNC outcomes in patients with AHRF admitted to the ICU in need of accurate medical demand, such as the COVID‐19 pandemic; however, our conclusion must be verified by a large-cohort and multicenter study in the future.

## Supplementary Information

**Additional file 1. Table S1:** Vital signs and parameters at the point of HFNC failure in 10 patients from the HFNC failure group. **Figure S1:** Relationship between FiO_2_ and the mROX index at PaO_2_ of 60 mmHg for respiratory rates (RRs) ranging between 20 and 40 bpm.

## Data Availability

The datasets used and/or analyzed during this study are available from the corresponding author on reasonable request.

## Abbreviations

AHRF: Acute hypoxemia respiratory failure
ICU: Intensive care unit
HFNC: High-flow nasal cannula
APACHE II: Acute physiology and chronical health evaluation II
HR: Heart rate
SpO_2_: Saturation of pulse oxygen
ROX: The respiratory rate oxygenation index
FiO_2_: Fraction of inspired oxygen
RR: Respiratory rate
MV: Mechanical ventilation
AUROC: Area under the receiver operating characteristic curve
GCS: Glasgow Coma Scale
