# Supplementary material for: Prediction of high-flow nasal cannula outcomes at the early phase using the modified respiratory rate oxygenation index
Source: BMC Pulm Med. 2022 Jun 13;22:227. doi: 10.1186/s12890-022-02017-8 (PMC9189451; doi:10.1186/s12890-022-02017-8)
Supplement: Supplementary file 1 — Additional file 1. Table S1: Vital signs and parameters at the point of HFNC failure in 10 patients from the HFNC failure group. Figure S1: Relationship between FiO2 and the mROX index at PaO2 of 60 mmHg for respiratory rates (RRs) ranging between 20 and 40 bpm. [file 12890_2022_2017_MOESM1_ESM.docx]

Supplementary materials：

**Table S1:** Vital signs and parameters at the point of HFNC failure in 10 patients from the HFNC failure group

**Figure S1：**Relationship between FiO2 and the mROX index at PaO2 of 60 mmHg for respiratory rates (RRs) ranging between 20 and 40 bpm.

Table S1 Vital signs and parameters at the point of HFNC failure in 10 patients from the HFNC failure group

| Vital signs and parameters | HFNC failure  (n = 10)* |
| --- | --- |
| FiO_2_ [*M*(*P*_25_，*P*_75_),%] | 0.73(0.62–0.83) |
| Flow on HFNC [*M*(*P*_25_，*P*_75_), L/min] | 52.5(50.0–60.0) |
| HR [*M*(*P*_25_，*P*_75_), bpm] | 108.5(76.0–124.0) |
| RR [*M*(*P*_25_，*P*_75_), cpm] | 23.0(17.5–29.8) |
| SpO_2_[mean ± SD, %] | 91.1±8.2 |
| PH [*M*(*P*_25_，*P*_75_)] | 7.40(7.36–7.46) |
| PaO_2_ [*M*(*P*_25_，*P*_75_), mmHg] | 57.0(48.8–68.0) |
| PaCO_2_ [mean ± SD, mmHg] | 45.8±11.5 |
| Hemodynamic Status [n]  cardiac arrest  arrhythmias  norepinephrine (over 0.1 μg/kg/min) | 0  3  2 |
| Glasgow Coma Scale (GCS) | 3 |
| Automatic Depuration Ability [n] | 3 |

Annotation: HR, heart rate; RR, respiratory rate; FiO_2_, fraction of inspiration O_2_; SpO_2_, saturation of pulse oxygen; PaO_2_, arterial oxygen partial pressure; PaCO_2_, arterial carbon dioxide partial pressure; PF ratio, PaO_2_/FiO_2_; cpm, counts per minute; bpm, beats per minute; P_25_, 25th percentile; P_75_, 75th percentile; HFNC, high-flow nasal cannula.*Several patients met multiple criteria which lead to total count was greater than 10.

Figure S1：Relationship between FiO2 and the mROX index at PaO2 of 60 mmHg for respiratory rates (RRs) ranging between 20 and 40 bpm.


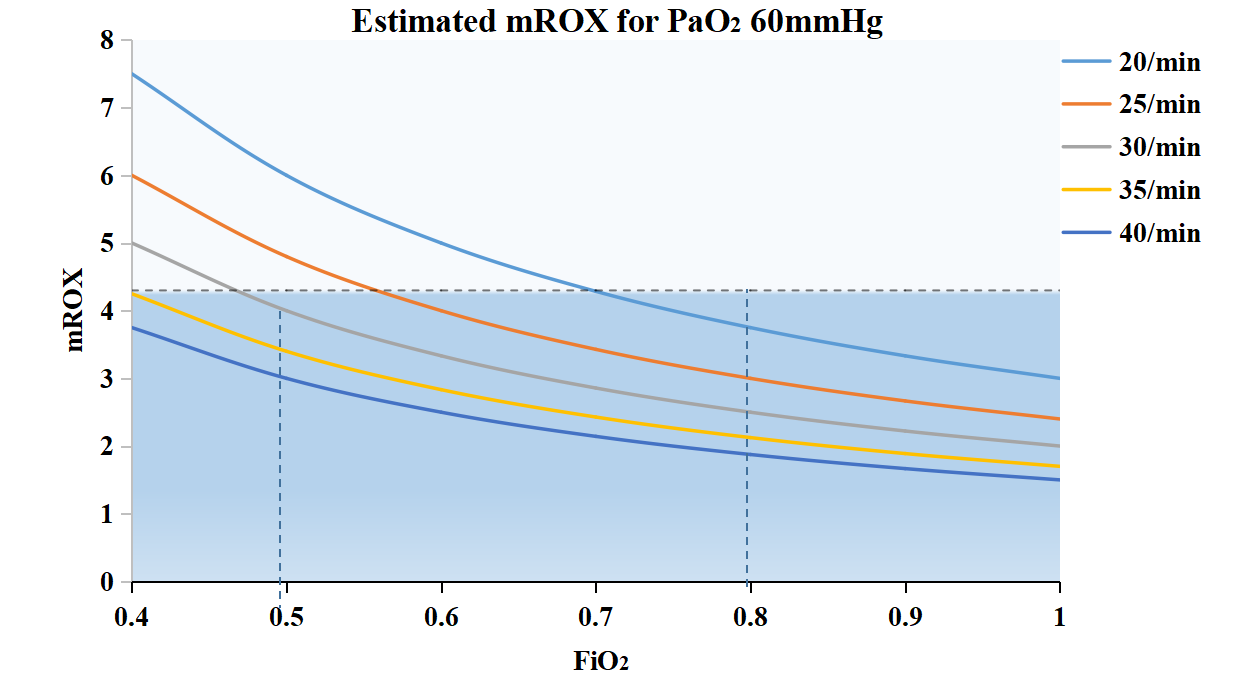


Figure S1. Relationship between FiO_2_ and the mROX index at PaO_2_ of 60 mmHg for respiratory rates (RRs) ranging between 20 and 40 bpm. The colored lines represent RR. The blue area indicates the mROX value; dashed vertical lines indicate FiO_2_ values of 0.5 and 0.8. mROX, ratio of PaO_2_/FiO_2_(%) to RR(breaths/min).
